# A low-cost hematologic biomarker for evaluating peripheral arterial disease risk: Derived neutrophil–lymphocyte ratio in NHANES 1999–2004 adults

**DOI:** 10.1097/MD.0000000000047010

**Published:** 2026-01-02

**Authors:** Qiang Liu, Xing Wu, Jianjun Shi

**Affiliations:** a Department of Cardiovascular Surgery, Taiyuan Central Hospital, Taiyuan, China; b Department of Cardiovascular Surgery, The Ninth Clinical College Affiliated to Shanxi Medical University, Taiyuan, China; c Department of Nephrology, Taiyuan Central Hospital, Taiyuan, China; d Department of Nephrology, The Ninth Clinical College Affiliated to Shanxi Medical University, Taiyuan, China.

**Keywords:** ankle-brachial index, cross-sectional study, derived neutrophil–lymphocyte ratio, National Health and Nutrition Examination Survey, peripheral arterial disease

## Abstract

Peripheral arterial disease (PAD), which affects over 200 million individuals globally, signifies systemic atherosclerosis with significant morbidity and healthcare burden. Chronic inflammation underpins its pathogenesis, and highlights the need for accessible biomarkers. The derived neutrophil–lymphocyte ratio (dNLR) offers a cost-effective inflammatory indicator, but remains underexplored for PAD association in population-based studies. This cross-sectional analysis utilized the 1999–2004 National Health and Nutrition Examination Survey (NHANES) data from 5223 adults aged ≥40 years. PAD was defined as an ankle-brachial index < 0.90. dNLR quartiles were established, and multivariable logistic regression was used to evaluate the dNLR-PAD relationship across progressively adjusted models: model 1 (unadjusted), model 2 (demographics/socioeconomic factors/physical activity), model 3 (model 2 + metabolic markers), and model 4 (model 3 + comorbidities). Subgroup analyses were used to assess consistency. Higher dNLR quartiles showed a significantly reduced PAD prevalence (*Q*1: 8.1% vs *Q*4: 4.8%; *P* = .004). After full adjustment (model 4), each unit increase in the dNLR conferred 11% greater PAD odds (odds ratio [OR]: 1.11; 95% confidence interval: 1.03–1.19; *P* = .006). Participants in the highest quartile (*Q*4) had a 49% elevated PAD risk versus *Q*1 (OR: 1.49; 95% confidence interval: 1.05–2.11; *P* = .025), demonstrating a dose–dependent trend (*P* trend = .015). Stratified analyses confirmed robust associations across demographics and clinical subgroups, with pronounced effects in Mexican–Americans (OR: 1.44). Elevated dNLR is independently associated with higher PAD prevalence in a representative US cohort, supporting its utility as an economical, routinely obtainable inflammatory biomarker for risk stratification. Prospective studies should validate the optimal dNLR thresholds and integrate nutritional indices to refine PAD risk paradigms.

## 1. Introduction

Peripheral arterial disease (PAD) is a critical atherosclerotic syndrome characterized by progressive lipid-rich plaque accumulation in the lower-extremity arteries.^[1]^ This pathophysiological continuum, driven by chronic inflammation, endothelial dysfunction, and oxidative stress, culminates in arterial stenosis, hypoperfusion, and ischemic tissue injury.^[2,3]^ Clinical manifestations span a broad spectrum, ranging from subclinical states and exertional symptoms to advanced disease featuring rest pain, tissue loss, and critical limb ischemia, requiring amputation.^[4,5]^ Anatomical involvement varies markedly, ranging from focal femoropopliteal lesions to diffuse multisegmental disease, with epidemiological factors including female sex, dyslipidemia, diabetes mellitus, and renal impairment modulating phenotypic expression.^[6,7]^ Globally, PAD affects >200 million individuals, with 8.5 million US adults aged ≥40 years diagnosed.^[8,9]^ This condition imposes substantial multidimensional burdens through revascularization/amputation costs, long-term disability expenditures, and profound functional impairment with reduced quality-adjusted life-years.^[10]^ This finding underscores the need for enhanced risk detection and modifiable pathway identification.

Chronic inflammation is a fundamental mechanism governing atherosclerotic initiation, progression, and plaque destabilization in PAD.^[11,12]^ Thus, cost-effective inflammatory biomarkers derived from routine hematology hold promise for risk stratification. The neutrophil–lymphocyte ratio (NLR), a composite indicator of systemic inflammation, reflects neutrophilia (innate immune activation) and lymphopenia (physiological stress). Elevated NLR consistently correlates with atherosclerosis severity, PAD prevalence, symptomatic burden, and adverse cardiovascular outcomes.^[13–15]^ Furthermore, the NLR and related indices have demonstrated prognostic value for all-cause and cardiovascular mortality in large, representative cohorts like National Health and Nutrition Examination Survey (NHANES), underscoring their utility in population health research.^[16–18]^

The derived neutrophil–lymphocyte ratio (dNLR) provides a pragmatic alternative that require only basic complete blood count parameters. While dNLR demonstrates prognostic utility comparable or superior to NLR in oncology and acute coronary syndromes,^[19,20]^ evidence within the PAD context remains scarce. Contemporary biomarker research predominantly prioritizes NLR or high-sensitivity C-reactive protein (hs-CRP),^[21–23]^ leaving a significant gap regarding dNLR’s role of the dNLR in peripheral vasculopathy. Critically, no prior study has leveraged the NHANES, a nationally representative database with standardized ankle-brachial index (ABI ≤ 0.9) assessments and comprehensive hematological profiling to investigate dNLR-PAD associations.

This cross-sectional study addresses this evidence gap by rigorously evaluating the independent relationship between dNLR and prevalent PAD within the NHANES cohort. Utilizing this epidemiological resource will elucidate dNLR’s viability as an accessible inflammatory indicator for community-based PAD risk stratification.

## 2. Materials and methods

### 2.1. Study population

This retrospective cross-sectional analysis used data from the 1999–2004 NHANES, a nationally representative surveillance program directed by the National Center for Health Statistics. The NHANES employs a complex multistage probability sampling design with stratification and clustering to generate population health estimates.^[24]^ All procedures were approved by the National Center for Health Statistics Institutional Review Board and informed consent was obtained prior to participant enrollment.^[25]^ Publicly accessible deidentified data are available from the Centers for Disease Control and Prevention (https://www.cdc.gov/nchs/nhanes/).

The analytical cohort was derived from 31,126 participants through sequential exclusion. We first restricted the sample to adults aged ≥40 years (n = 9970) because ABI assessment was not conducted in younger populations. Subsequent exclusions included individuals with incomplete bilateral ABI measurements (n = 2399); those exhibiting ABI values > 1.4 (n = 73), indicating non-compressible vessels; participants lacking white blood cell (WBC), lymphocyte, or neutrophil counts (n = 1282); and subjects with missing covariate data (n = 993). These exclusions yielded a final analytical sample of 5223 participants with complete demographic, clinical, and hematological profiles.

### 2.2. Peripheral artery disease

ABI measurements were performed exclusively for participants aged ≥40 years according to standardized vascular evaluation protocols. Following a mandatory 5-minute supine rest period, oscillometric systolic blood pressure (SBP) recordings were systematically obtained initially from the right brachial artery (with left brachial measurement substituted when clinically indicated), followed by bilateral assessments of the posterior tibial and dorsalis pedis arteries. To ensure methodological consistency across age groups, participants aged 40 to 59 years underwent duplicate measurements at all sites with subsequent averaging, whereas those aged ≥60 years received single measurements per vascular territory.

ABI computation involved dividing the mean ankle SBP (averaged across both lower extremities) by the brachial SBP value. The final disease classification was determined by using the lowest extremity-specific ABI value. Consistent with international consensus guidelines, PAD diagnosis was operationally defined as ABI < 0.90 in either lower limb.^[26,27]^ This protocol accounted for age-specific measurement requirements, while maintaining a standardized perfusion assessment across anatomical variations.

### 2.3. Study variables and outcome

The dNLR is a hematological index calculated as the neutrophil count divided by the difference between the WBC and lymphocyte counts.^[28,29]^

Sociodemographic and lifestyle variables were ascertained through structured NHANES questionnaires, while clinical measurements, including anthropometric indices, hemodynamic parameters, and biochemical analysis, were obtained during Mobile Examination Center assessments. Informed by epidemiological evidence,^[30–33]^ covariates encompassed demographic factors (age, gender, ethnicity, education, marital status), socioeconomic indicators (family income categorized by poverty-income ratio: low ≤ 1.3, medium 1.3–3.5, high > 3.5), behavioral determinants (physical activity intensity: below moderate [no ≥10-minutes bouts], moderate [exclusively moderate activities], high [any vigorous activity]), biometric parameters (body mass index [BMI], total cholesterol, hs-CRP, glycosylated hemoglobin [HbA1c]), and clinical comorbidities operationalized per consensus standards: hypertension (SBP ≥ 140 mm Hg/diastolic BP ≥ 90 mm Hg, antihypertensives, or diagnosis), diabetes (fasting glucose ≥ 7.0 mmol/L, HbA1c ≥ 6.5%, hypoglycemic agents, or physician-confirmed history), and cardiovascular disease (self-reported heart failure, coronary artery disease, angina, myocardial infarction, or stroke) – with anthropometric methodologies detailed in NHANES technical documentation (https://www.cdc.gov/nchs/nhanes/).

### 2.4. Statistical analysis

Categorical variables are expressed as proportions (%) and continuous variables as mean ± (stdard deviation) or median (interquartile range). Group comparisons were performed using ANOVA, Kruskal–Wallis, or χ² tests based on data distribution characteristics. This secondary analysis utilized de-identified NHANES data without incorporating sampling weights or complex survey design elements to prioritize internal validity for pathophysiological inference over population generalizability, acknowledging the potential limitations in extrapolation while maintaining statistical parsimony.

dNLR-PAD associations were evaluated using 5 progressively adjusted logistic regression models: model 1 (unadjusted), model 2 (demographics + socioeconomic status + physical activity), model 3 (model 2 + metabolic factors), and model 4 (model 3 + clinical comorbidities). Covariates were selected based on clinical relevance, literature, and effect modification thresholds (>10% outcome influence). This hierarchical approach generated adjusted odds ratios (aORs) with 95% confidence interval [CIs], systematically isolating dNLR’s inflammatory effects of the dNLR while controlling for demographic, behavioral, metabolic, and clinical confounders.

Stratified analyses assessed association consistency across demographic (age, sex), socio-behavioral (activity, income, education), and clinical subgroups (CVD, hypertension, and diabetes status), with interaction effects quantified using multivariable logistic regression and likelihood ratio testing. All analyses were conducted using R Statistical Software (V4.2.2, http://www.R-project.org, The R Foundation) and a Free Statistics Analysis Platform (V1.9, Beijing, China, http://www.clinicalscientists.cn/freestatistics), with comprehensive methodological documentation ensuring transparency and reproducibility throughout the analytical workflow.

## 3. Results

### 3.1. Characteristics of the study population

**Figure 1.**
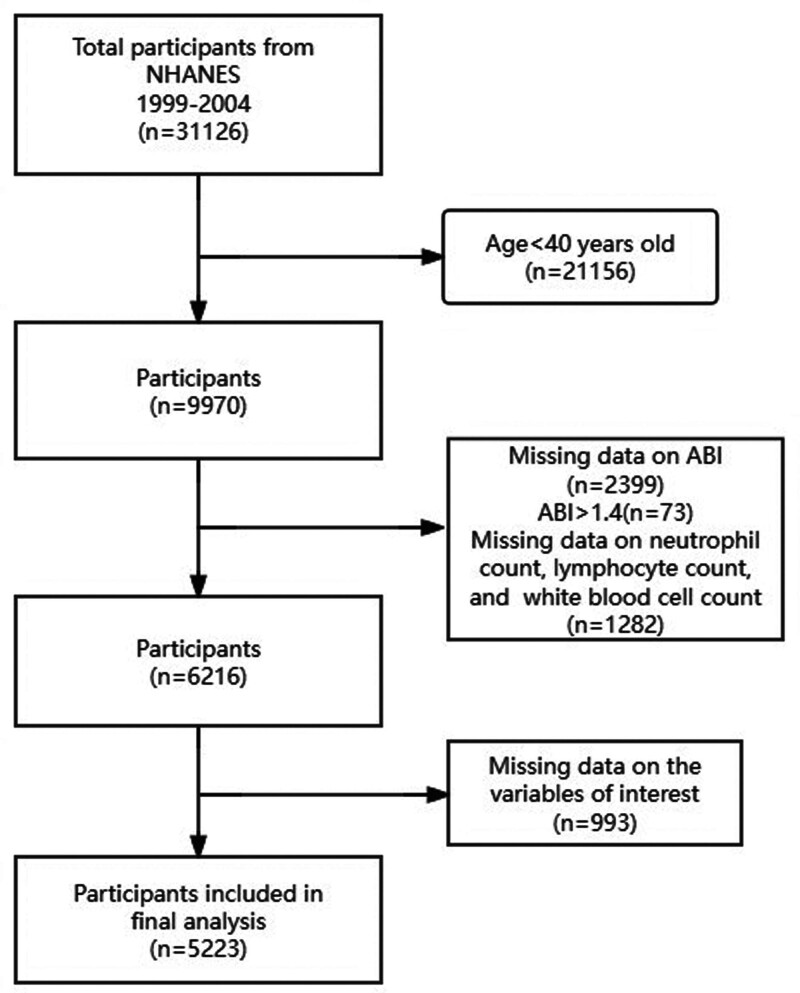
Flow chart of participant selection from the NHANES 1999–2004 database. NHANES = National Health and Nutrition Examination Survey.

The analytical cohort comprised 5223 participants, following sequential exclusions from an initial pool of 31,126 respondents who completed in-home interviews (Fig. 1). Table 1 displays the baseline characteristics of the participants stratified by dNLR quartile. Significant differences were observed across quartiles. Notably, PAD prevalence decreased with higher dNLR quartiles (*Q*1: 8.1% vs *Q*4: 4.8%; *P* = .004). Participants in higher quartiles were younger (*Q*4: 58.9 ± 12.6 years vs *Q*2: 60.8 ± 13.2; *P* = .002) and had lower BMI (*Q*4: 27.9 ± 5.4 kg/m² vs *Q*1: 28.8 ± 5.9; *P* < .001). Striking racial distribution shifts occurred, with declining non-Hispanic White representation (*Q*1: 58.5% → *Q*4: 46.5%) and increasing non-Hispanic Black representation (*Q*1: 12.3% → *Q*4: 28.7%; *P* < .001). Higher dNLR quartiles were correlated with improved metabolic profiles: lower CRP (median *Q*4: 0.2 vs *Q*1: 0.3; *P* < .001), reduced HbA1c (*Q*4: 5.7 ± 1.0% vs *Q*1: 5.9 ± 1.2%; *P* = .002), and decreased hypertension/diabetes prevalence (both *P* < .05). Physical activity levels increased (*P* < .001), lymphocyte counts decreased, and neutrophil counts increased monotonically across quartiles (both *P* < .001).

**Table 1:** Baseline characteristics stratified by the derived neutrophil–lymphocyte ratio (dNLR) quartiles (*Q*).

Variables	Total	dNLR				*P*-value
	(n = 5223)	*Q*1 (n = 1306)	*Q*2 (n = 1305)	*Q*3 (n = 1306)	*Q*4 (n = 1306)	
Gender, n (%)						.846
Male	2685 (51.4)	660 (50.5)	681 (52.2)	668 (51.1)	676 (51.8)	
Female	2538 (48.6)	646 (49.5)	624 (47.8)	638 (48.9)	630 (48.2)	
Age (yr), mean ± SD	60.0 ± 13.0	59.9 ± 13.0	60.8 ± 13.2	60.2 ± 12.9	58.9 ± 12.6	.002
Race, n (%)						<.001
Mexican American	1070 (20.5)	295 (22.6)	262 (20.1)	261 (20)	252 (19.3)	
Other Hispanic	203 (3.9)	53 (4.1)	60 (4.6)	50 (3.8)	40 (3.1)	
Non-Hispanic White	2903 (55.6)	764 (58.5)	778 (59.6)	754 (57.7)	607 (46.5)	
Non-Hispanic Black	901 (17.3)	161 (12.3)	166 (12.7)	199 (15.2)	375 (28.7)	
Other races	146 (2.8)	33 (2.5)	39 (3)	42 (3.2)	32 (2.5)	
BMI, kg/m^2^, mean ± SD	28.4 ± 5.6	28.8 ± 5.9	28.5 ± 5.5	28.5 ± 5.6	27.9 ± 5.4	<.001
Total cholesterol, mg/dL, mean ± SD	208.8 ± 40.1	208.7 ± 40.4	210.3 ± 39.4	210.1 ± 40.4	206.1 ± 39.9	.025
CRP, median (IQR)	0.2 (0.1, 0.5)	0.3 (0.1, 0.7)	0.3 (0.1, 0.6)	0.2 (0.1, 0.5)	0.2 (0.1, 0.4)	<.001
HbA1c, %	5.8 ± 1.1	5.9 ± 1.2	5.8 ± 1.1	5.8 ± 1.2	5.7 ± 1.0	.002
Education level, n (%)						.005
Less than high school	907 (17.4)	246 (18.8)	230 (17.6)	223 (17.1)	208 (15.9)	
High school diploma or GED	2011 (38.5)	526 (40.3)	527 (40.4)	491 (37.6)	467 (35.8)	
More than high school	2305 (44.1)	534 (40.9)	548 (42)	592 (45.3)	631 (48.3)	
MS, n (%)						.152
Married/living with partner	3480 (66.6)	849 (65)	867 (66.4)	888 (68)	876 (67.1)	
Widowed/divorced/separated	1449 (27.7)	379 (29)	378 (29)	349 (26.7)	343 (26.3)	
Never married	294 (5.6)	78 (6)	60 (4.6)	69 (5.3)	87 (6.7)	
PIR, n (%)						.122
Low income	1303 (24.9)	355 (27.2)	320 (24.5)	317 (24.3)	311 (23.8)	
Medium income	1964 (37.6)	507 (38.8)	486 (37.2)	488 (37.4)	483 (37)	
High income	1956 (37.4)	444 (34)	499 (38.2)	501 (38.4)	512 (39.2)	
Hypertension, n (%)						<.001
No	3370 (64.5)	791 (60.6)	828 (63.4)	872 (66.8)	879 (67.3)	
Yes	1853 (35.5)	515 (39.4)	477 (36.6)	434 (33.2)	427 (32.7)	
Diabetes, n (%)						.038
No	4556 (87.2)	1115 (85.4)	1130 (86.6)	1151 (88.1)	1160 (88.8)	
Yes	667 (12.8)	191 (14.6)	175 (13.4)	155 (11.9)	146 (11.2)	
Physical activity, n (%)						<.001
Sedentary	2377 (45.5)	647 (49.5)	583 (44.7)	598 (45.8)	549 (42)	
Moderate	1638 (31.4)	397 (30.4)	449 (34.4)	389 (29.8)	403 (30.9)	
Vigorous	1208 (23.1)	262 (20.1)	273 (20.9)	319 (24.4)	354 (27.1)	
CVD, n (%)						<.001
No	4421 (84.6)	1068 (81.8)	1096 (84)	1106 (84.7)	1151 (88.1)	
Yes	802 (15.4)	238 (18.2)	209 (16)	200 (15.3)	155 (11.9)	
Lymphocyte count, median (IQR)	2.0 (1.6, 2.5)	2.1 (1.7, 2.7)	2.0 (1.6, 2.5)	1.9 (1.6, 2.4)	1.8 (1.5, 2.3)	<.001
Neutrophil count, mean ± SD	4.1 ± 1.8	2.5 ± 0.8	3.5 ± 0.9	4.4 ± 1.2	5.9 ± 1.9	<.001
White blood cell count, mean ± SD	7.1 ± 2.5	8.6 ± 3.6	7.4 ± 1.8	6.7 ± 1.5	5.6 ± 1.4	<.001
PAD, n (%)						.004
No	4900 (93.8)	1200 (91.9)	1224 (93.8)	1233 (94.4)	1243 (95.2)	
Yes	323 (6.2)	106 (8.1)	81 (6.2)	73 (5.6)	63 (4.8)	

*Q* values are presented as mean ± standard deviation, median (IQR), or numbers and percentages.

BMI = body mass index, CRP = C-reactive protein, CVD = cardiovascular disease, dNLR = derived neutrophil-lymphocyte ratio, GED = general educational development, HbA1c = glycosylated hemoglobin, MS = marital status, PAD = peripheral arterial disease, PIR = poverty-income ratio, *Q* = quartile, SD = standard deviation.

### 3.2. Association between dNLR and PAD

Univariate analysis identified significant associations of age, marital status, family income, physical activity level, education level, total cholesterol, CRP levels, lymphocyte count, WBC count, neutrophil count, HbA1c, prevalent CVD, hypertension, and diabetes (Table 2).

**Table 2:** Association of covariates and PAD risk.

Variable	OR (95% CI)	*P*-value
Gender, n (%)		
Male	1 (reference)	
Female	1.11 (0.89–1.39)	.355
Age (yr), mean ± SD	1.08 (1.07–1.09)	<.001
Race, n (%)		
Mexican American	1 (reference)	
Other Hispanic	0.97 (0.47–2)	.927
Non-Hispanic White	1.39 (1.01–1.93)	.044
Non-Hispanic Black	1.97 (1.37–2.86)	<.001
Other races	0.74 (0.29–1.88)	.526
BMI, kg/m^2^, mean ± SD	0.99 (0.97–1.01)	.167
Total cholesterol, mg/dL, mean ± SD	1 (1–1)	.289
CRP, median (IQR)	1.26 (1.14–1.38)	<.001
HbA1c, %	1.22 (1.14–1.31)	<.001
Education level, n (%)		
Less than high school	1 (reference)	
High school diploma or GED	0.76 (0.57–1.01)	.057
More than high school	0.48 (0.35–0.65)	<.001
MS, n (%)		
Married/living with partner	1 (reference)	
Widowed/divorced/separated	2.11 (1.67–2.65)	<.001
Never married	0.54 (0.26–1.1)	.091
PIR, n (%)		
Low income	1 (reference)	
Medium income	0.86 (0.67–1.12)	.276
High income	0.45 (0.33–0.6)	<.001
Hypertension, n (%)		
No	1 (reference)	
Yes	2.9 (2.3–3.65)	<.001
Diabetes, n (%)		
No	1 (reference)	
Yes	2.6 (2–3.39)	<.001
Physical activity, n (%)		
Sedentary	1 (reference)	
Moderate	0.62 (0.48–0.8)	<.001
Vigorous	0.28 (0.19–0.41)	<.001
CVD, n (%)		
No	1 (reference)	
Yes	3.24 (2.54–4.13)	<.001
Lymphocyte count, median (IQR)	1 (0.94–1.07)	.921
Neutrophil count, mean ± SD	1.02 (0.96–1.09)	.473
White blood cell count, mean ± SD	1.08 (1.04–1.13)	<.001
dNLR	0.69 (0.54–0.88)	<.001

BMI = body mass index, CI = confidence interval, CRP = C-reactive protein, CVD = cardiovascular disease, dNLR = derived neutrophil-to-lymphocyte ratio, GED = general educational development, HbA1c = glycosylated hemoglobin, IQR = interquartile range, MS = marital status, PIR = poverty-income ratio, PAD = peripheral arterial disease, OR = odds ratio, SD = standard deviation.

Table 3 demonstrates a significant positive association between dNLR and PAD risk. In the fully adjusted model (model 4), each unit increase in the dNLR conferred 11% higher PAD odds (OR: 1.11, 95% CI: 1.03–1.19; *P* = .006). Crucially, participants in the highest dNLR quartile (*Q*4: 2.16–17.75) exhibited a substantially elevated risk versus *Q*1 reference (OR: 1.49, 95% CI: 1.05–2.11; *P* = .025). A consistent dose–response relationship was observed across all models (*P*-trend ≤ .015), with *Q*4 risk estimates ranging from OR 1.79 (model 1, *P* < .001) to OR 1.49 (model 4). Notably, this positive association persisted after sequential adjustments for sociodemographic factors, metabolic biomarkers, and cardiovascular comorbidities.

**Table 3:** Association between dNLR and PAD.

Variable	dNLR	*P*-value	*Q*1 (−9.66 to 0.70)	*Q*2 (0.71–1.46)	*P*-value	*Q*3 (1.47–2.15)	*P*-value	*Q*4 (2.16–17.75)	*P*-value	Trend test	*P*-value
	OR (95% CI)		OR (95% CI)	OR (95% CI)		OR (95% CI)		OR (95% CI)			
Model 1	1.18 (1.10–1.27)	<.001	1 (Ref)	1.05 (0.74–1.49)	.786	1.21 (0.86–1.70)	.264	1.79 (1.31–2.64)	<.001	1.22 (1.10–1.35)	<.001
Model 2	1.15 (1.07–1.24)	<.001	1 (Ref)	1.10 (0.76–1.59)	.603	1.26 (0.88–1.80)	.204	1.75 (1.25–2.46)	.001	1.21 (1.08–1.35)	.001
Model 3	1.13 (1.05–1.22)	.001	1 (Ref)	1.07 (0.74–1.55)	.712	1.20 (0.84–1.72)	.320	1.61 (1.14–2.28)	.006	1.18 (1.05–1.31)	.004
Model 4	1.11 (1.03–1.19)	.006	1 (Ref)	1.05 (0.73–1.53)	.780	1.21 (0.84–1.73)	.312	1.49 (1.05–2.11)	.025	1.15 (1.03–1.28)	.015

Model 1 adjusted for none.

Model 2 adjusted for age + sex + race + education level + MS + PIR + physical activity.

Model 3 was adjusted for model 2 + BMI + total cholesterol + CRP + HbA1c.

Model 4 adjusted for model 3 + cardiovascular disease + hypertension + diabetes.

BMI = body mass index, CRP = C-reactive protein, dNLR = derived neutrophil-to-lymphocyte ratio, HbA1c = glycosylated hemoglobin, MS = marital status, PIR = poverty-income ratio, PAD = peripheral arterial disease, *Q* = quartile.

### 3.3. Stratification analysis

**Figure 2.**
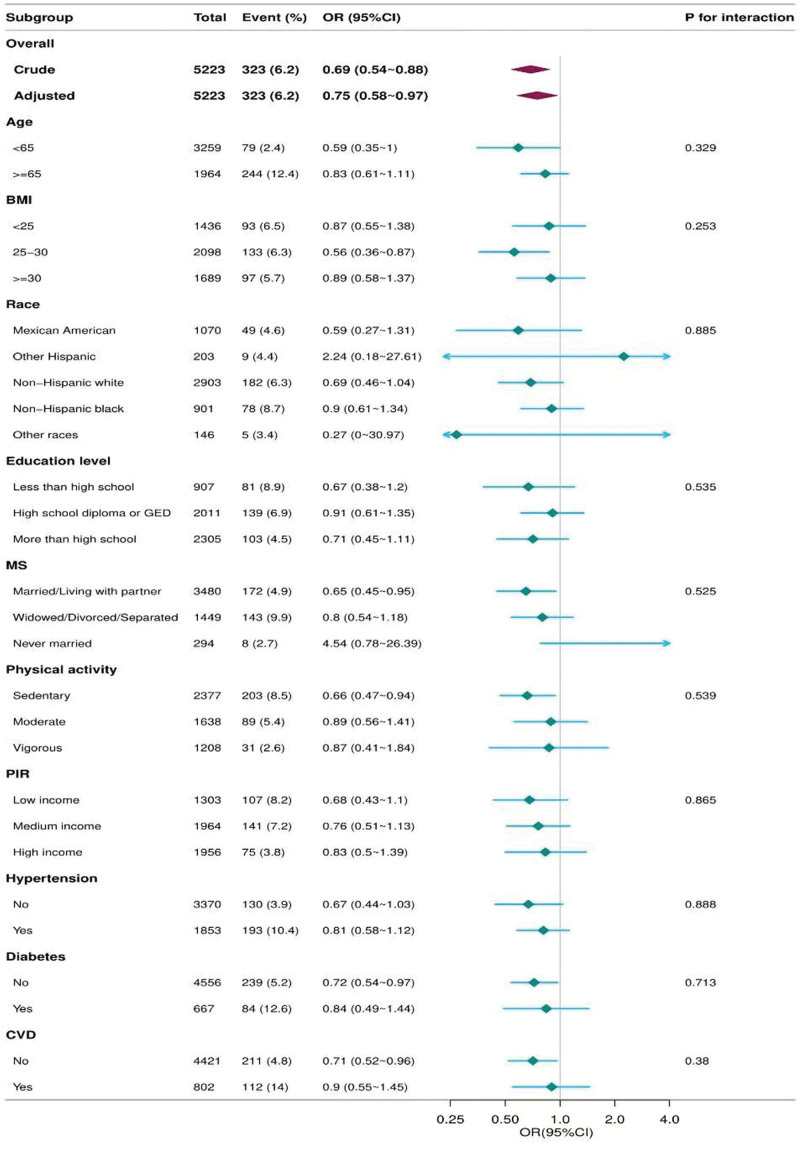
Forest plot showing the association between dNLR and PAD risk across subgroups. CI = confidence interval, dNLR = derived neutrophil-to-lymphocyte ratio, OR = odds ratio, PAD = peripheral arterial disease.

Forest plot analysis confirmed a consistent positive association between dNLR and PAD risk across all subgroups. Each unit increase in dNLR increased the adjusted PAD risk by 11% (OR: 1.11, 95% CI: 1.03–1.19). Notably, stronger effects emerged in Mexican Americans (OR = 1.44) and married individuals (OR = 1.28). This association persisted irrespective of comorbidities, with significant effects in the normotensive (OR: 1.19), diabetic (OR: 1.13), and non-diabetic (OR: 1.19) subgroups. The absence of interaction effects (all *P* > .05) and consistent risk elevation across age, BMI, and activity strata establish the dNLR as a universal biomarker for PAD risk assessment (Fig. 2).

## 4. Discussion

This study established a significant positive association between the dNLR and PAD prevalence in a nationally representative cohort. We observed a dose–dependent risk elevation across ascending dNLR quartiles, which persiste after a comprehensive adjustment for cardiovascular and metabolic confounders. Subgroup analyses confirmed robust associations across demographic strata, with Mexican–Americans exhibiting heightened susceptibility. The restricted cubic spline analysis revealed a linear relationship without threshold effects.

Our findings align with contemporary evidence on dNLR’s vascular implications of dNLR. Belaj et al reported a stepwise increase in the incidence of critical limb ischemia across dNLR tertiles (20.4% → 26.1% → 36.1%; *P* < .001 by Jonckheere–Terpstra test),^[34]^ while Fan et al demonstrated that elevated dNLR independently predicted major adverse cardiovascular events post-PCI (HR: 2.610, 95% CI: 1.454–4.685; *P* = .001).^[20]^ The dose–response relationship observed in our cohort mirrors García-Rivera et al’s documentation of dNLR-associated amputation risk in critical limb-threatening ischemia (NLR: 6.91 ± 7.85 vs 3.94 ± 2.57 in amputation vs non-amputation groups; *P* = .023).^[35]^ In contrast with Ma et al’s threshold effect for mortality outcomes (log-dNLR > 0.370; CV mortality HR: 1.38, 95% CI: 1.14–1.68),^[36]^ our linear association with prevalent PAD likely reflects methodological differences, including endpoint selection (prevalent disease vs mortality), analytical approach (log-transformation), and ethnic susceptibility patterns.^[37]^ The amplified risk in Mexican Americans (OR: 1.44) aligns with Bath et al’s report of racial disparities in NLR-severity gradients (tissue loss prevalence: 20.9% Black vs 12.9% White; *P* < .0001).^[22]^

The dNLR demonstrates superior stability versus the conventional NLR across BMI strata (all ORs > 1.15), whereas the traditional NLR exhibits severity-dependent variability.^[38,39]^ This advantage is further corroborated by meta-analytic evidence showing dNLR’s predictive consistency of the dNLR for 1-year mortality (AUC, 0.71; 95% CI: 0.59–0.79).^[37]^ The prognostic utility of the biomarker extends beyond peripheral disease, predicting post-PCI mortality in coronary pathology (dNLR ≥ 1.96 tertile: all-cause mortality HR: 1.763, 95% CI: 1.133–2.743; *P* = .012),^[40]^ and enabling composite risk scores such as the dNLR-PNI system (HR: 3.049, 95% CI: 1.503–6.184; *P* = .002 for MACE).^[41]^

Emerging applications highlight dNLR’s versatility of the dNLR, including the prediction of post-stroke infections and correlation with stroke severity biomarkers.^[39]^ Nevertheless, NLR retains value for perioperative complication assessment (e.g., amputation OR: 2.5, 95% CI: 1.65–3.87 in the high NLR group),^[22]^ underscoring context-dependent utility. Our comprehensive adjustment for socioeconomic confounders addresses a recognized literature gap,^[42]^ while recent work on inflammatory synergism shows direct correlations between NLR/PLR and stenotic lesion burden (*P* = .018 and *P* = .016, respectively).^[38]^ The utility of dNLR in PAD risk assessment resonates with the emerging paradigm of multi-component biomarkers in cardiovascular medicine. Supporting this approach, recent work by Cheng et al establishes a joint association of the neutrophil-to-lymphocyte ratio and the atherogenic index of plasma with incident cardiovascular disease.^[16]^ These findings illuminate a promising path for refining PAD risk stratification by amalgamating inflammatory indices such as dNLR with metabolic profiles, potentially yielding superior predictive models for atherosclerosis.

The linear dNLR-PAD association reflects the cumulative vascular injury mediated through neutrophil-dominated pathways. This cumulative injury manifests clinically as amputation risk, where the dNLR’s correlation with stenotic lesion burden^[43]^ parallels NLR-based revascularization failure prediction.^[15]^ Sustained neutrophilia promotes neutrophil extracellular trap propagation, driving arterial thrombosis,^[44]^ whereas chronic inflammation induces lymphoid exhaustion that compromises vascular homeostasis.^[45]^ NLRP3 inflammasome activation further exacerbates endothelial dysfunction.^[46]^ The dNLR formula enhances detection sensitivity by incorporating granulocyte precursors that are often underrepresented in conventional ratios,^[47]^ correlating clinically with endothelial damage biomarkers that establish a prothrombotic milieu.

Key strengths include NHANES’s standardized protocols and novel covariate adjustments. Limitations warrant consideration: unweighted analysis potentially biases minority subgroup estimates^[26]^; cross-sectional design precludes causal inference; single dNLR measurements overlook circadian variation; and the absence of advanced vascular imaging restricts severity stratification.

## 5. Conclusions

The dNLR demonstrated strong utility as an accessible inflammatory biomarker for PAD risk assessment. Future studies should prospectively validate optimal thresholds and explore their integration with nutritional indices to enhance risk stratification paradigms.

## Acknowledgments

We express our gratitude to Jie Liu from the Department of Vascular and Endovascular Surgery at the Chinese PLA General Hospital for providing valuable assistance in statistical analysis, study design consultations, and insightful feedback on the manuscript.

## Author contributions

**Conceptualization:** Qiang Liu, Xing Wu.

**Data curation:** Xing Wu.

**Formal analysis:** Xing Wu.

**Funding acquisition:** Jianjun Shi.

**Methodology:** Xing Wu.

**Resources:** Jianjun Shi.

**Supervision:** Qiang Liu.

**Validation:** Qiang Liu.

**Visualization:** Qiang Liu, Jianjun Shi.

**Writing – original draft:** Qiang Liu, Xing Wu.

**Writing – review & editing:** Jianjun Shi.
